# Optimization of Ni-B-Mo Electroless Coating on GCr15 Steel: Effects of Main Salt Concentration and Deposition Time

**DOI:** 10.3390/ma18091981

**Published:** 2025-04-27

**Authors:** Shunqi Mei, Xiaohui Zou, Zekui Hu, Jinyu Yang, Quan Zheng, Wei Huang, Alexey Guryev, Burial Lygdenov

**Affiliations:** 1 Hubei Digital Textile Equipment Key Laboratory, Wuhan Textile University, Wuhan 430073, China; sqmei@wtu.edu.cn (S.M.);; 2 The Advanced Textile Technology Innovation Center (Jianhu Laboratory), Shaoxing 312000, China; 3 School of Mechanical & Electrical Engineering, Xi’an Polytechnic University, Xi’an 710048, China; 4 Faculty of Chemistry, National Research Tomsk State University, Lenin Ave., 36, 634050 Tomsk, Russia; 5 Zhejiang Pinnuo Machinery Co., Ltd., Shaoxing 312500, China; gurievam@mail.ru; 6 Zhejiang Tianxiong Industrial Technology Co., Ltd., Shaoxing 312500, China; lygdenov59@mail.ru

**Keywords:** electroless Ni-B-Mo coating, GCr15 steel, hardness, wear resistance, adhesion

## Abstract

GCr15 bearing steel is widely used in the textile, aerospace, and other industries due to its excellent mechanical properties. However, traditional electroless Ni-B coatings can no longer meet the growing demand for a long service life under high-speed and heavy load conditions. This study focused on depositing Ni-B-Mo alloy coatings on GCr15 steel. An orthogonal experimental design was adopted to investigate the effects of the NiCl_2_ and Na_2_MoO_4_ concentrations and deposition time on the deposition rate and surface hardness of the coatings. The results show that the Na_2_MoO_4_ concentration has the most significant impact on the deposition rate. An optimal concentration of 5.6 g/L improved both the deposition rate and hardness (up to 881 HV), while excessive Na_2_MoO_4_ (>15.6 g/L) reduced the coating adhesion and wear resistance. A deposition time of 1–2 h ensured a high deposition rate, but after 3 h, bath component depletion lowered the rate and caused coating defects. The NiCl_2_ concentration (20–30 g/L) had a relatively minor influence on the deposition rate but stabilized the Ni^2+^ ion supply, enhancing the coating compactness. The optimized parameters were 5.6 g/L Na_2_MoO_4_, 25 g/L NiCl_2_, and 2 h of deposition. The coating exhibited high hardness, strong adhesion, and excellent wear resistance. After heat treatment at 400 °C for 1 h, the coating transitioned from being amorphous to nanocrystalline, forming Ni_2_B, Ni_3_B, and Mo_2_C phases, increasing the hardness from 737.49 HV to 916.19 HV and reducing the friction coefficient to 0.38.

## 1. Introduction

GCr15 steel is one of the most representative alloy steels among bearing alloys, known for its excellent comprehensive properties [1]. It is widely used in industries such as textiles, aerospace, automotives, and marine engineering, primarily for manufacturing critical rotating components, including steel balls, rollers, and bushings in drive shafts [2]. However, due to its specific operating environments and working conditions, the surface of GCr15 steel is prone to wear and corrosion, leading to performance degradation and an increased failure rate during service [3]. Different ways to treat surfaces, such as carburizing, nitriding, laser surface quenching, and physical vapor deposition, are commonly used to enhance the surface properties of GCr15 steel [4]. Electroless plating has emerged as a crucial surface modification technique due to its unique advantages. Unlike electroplating, electroless deposition does not require an external electric current but relies on chemical reactions to uniformly deposit metal or alloy coatings onto the substrate surface [5]. This method offers benefits such as a uniform coating distribution, high hardness, superior corrosion resistance, and suitability for complex-shaped components [6].

In electroless plating research, Ni-P coatings are widely used in marine engineering and chemical equipment due to their excellent corrosion resistance [7]. However, their relatively low hardness and wear resistance limit their applicability under high-load conditions. To address this limitation, researchers have developed Ni-B coatings. Sankara Narayanan and others used a special nickel coating method employing borohydride on copper, AISI 304 stainless steel, and medium-carbon steel, which greatly improved the coating’s hardness and ability to resist wear [8]. Baskaran et al. [9] created Ni-B coatings on copper and AISI 304 stainless steel using a low-temperature (45 °C) electroless plating bath that was reduced with borohydride. Their study revealed that the deposition rate, boron content, and magnetic properties of the coatings were dependent on the NaBH_4_ concentration. At a low NaBH_4_ concentration (0.4 g/L), the deposited coating exhibited a crystalline phase, whereas at a high NaBH_4_ concentration (1.0 g/L), the coating presented a mixed crystalline–amorphous structure. Oraon and others [10] improved the process settings for applying electroless Ni-B coatings on very pure copper (99.99%) using a response surface methodology, resulting in a hardness of 1171 ± 27 HV (11.5 ± 0.26 GPa) after the coating was applied. Hamid et al. [11] studied how to apply Ni-B coatings on low-carbon steel and 99.99% pure copper using DMAB as a reducing agent in a cold acidic solution. Their findings indicated that the deposition rate was temperature-dependent, while heat treatment enhanced the coating’s hardness, adhesion, and oxidation resistance. Notably, coatings annealed at 400 °C exhibited the lowest corrosion rate due to the formation of the Ni_3_B phase.

However, the performance of Ni-B coatings in corrosive environments remains inadequate, limiting their broader application [12]. To further enhance coating properties, researchers have introduced molybdenum (Mo), leading to the development of Ni-B-Mo alloy coating systems. Drovosekov and colleagues [13] looked into applying Ni-B-Mo coatings on nickel and copper using a chemical process that incorporates a pyrophosphate and DMB. Their study showed that the amount of TEA (a surfactant) had a big effect on both the Mo content and how quickly the coating was applied: lower amounts sped up the coating process, while higher amounts lowered the Mo content in the coating. Serin et al. [14] analyzed the tribological behavior of Ni-B-Mo coatings on low-carbon steel under different heat treatment conditions. Their results demonstrated that the coatings exhibited a uniform surface morphology and excellent compatibility with the steel substrate. Increasing the annealing temperature enhanced corrosion resistance, with the optimal performance observed at 550 °C. They also found that the wear resistance was lowest in the as-deposited state, peaked after annealing at 300 °C, remained nearly unchanged at 450 °C, and slightly decreased at 550 °C [15]. Mukhopadhyay et al. [16] studied how different heating temperatures affected the hardness and wear performance of electroless Ni-B-Mo coatings on AISI 1040 steel. Their findings indicated that heat-treated coatings exhibited good thermal stability at elevated temperatures, with a minimal change in the wear rate and a stable coefficient of friction. At 500 °C, a protective oxide layer formed on the worn surface. Additionally, microstructural changes, induced by high-temperature exposure and influenced by wear debris, affected the tribological mechanisms of the coating. Barman et al. [17] studied Ni-B-Mo alloy coatings on AISI 1040 steel under different bath compositions. Their results indicated that the coating thickness increased with higher borohydride concentrations. Increasing the Mo concentration led to a rise in the surface roughness (from 0.28 μm to 1.05 μm Ra) and an increase in the coefficient of friction (from 0.24 to 0.77). Even though adding more boron made the coating harder (an increase from 530 HV to 971 HV), adding Mo caused both the hardness and scratch resistance to decrease.

In short, many studies on electroless Ni-B-Mo coatings have examined how factors like the temperature, pH levels, and different reducing agents (such as NaBH_4_ and DMAB) influence the coating properties, especially on materials like AISI 1040 steel and copper. However, there are still several crucial issues with Ni-B-Mo coatings that need further investigation. Firstly, analysis indicates that the main salt concentration and deposition time are crucial parameters influencing the coating quality in electroless plating [18]. However, most studies have overlooked their impact on the coating structure and properties, making it challenging to optimize the deposition process. Secondly, changes in the main salt concentration can influence how much Mo is deposited, along with other materials, and the types of solid forms that result. The deposition mechanism of Mo within the Ni matrix requires further investigation. Additionally, most studies on Ni-B-Mo coatings have focused on mild steel or low-alloy steel, while their application on high-carbon chromium-bearing steel (GCr15) remains limited. The deposition mechanism of these coatings on GCr15 steel has yet to be fully elucidated. Moreover, the existing research often focuses on individual properties (e.g., wear resistance or corrosion resistance), lacking a comprehensive analysis of the coatings’ overall performance under optimized conditions.

Therefore, this study focused on the effects of different main salt concentrations and deposition times on Ni-B-Mo coatings. Using GCr15 steel as the base material, we carefully studied how changing the main salt concentration affects the way Mo is added to the coating. Additionally, the influence of the deposition time on the coating thickness, microstructure, and wear resistance was analyzed to determine the optimal plating process.

## 2. Materials and Methods

### 2.1. Materials

Table 1 details the chemical composition of the annealed GCr15 steel plates, which served as the substrate for electroless Ni-B-Mo deposition. The steel plates were cut into specimens of 20 × 15 × 5 mm^3^ using a DK7740 (Jiangsu Taizhou Chuangyuan Machine Tool Co., Taizhou, China) wire cutting electrical discharge machine. We drilled a hole at one end of each specimen to enable suspension from a hanging wire.

### 2.2. Preparation of Ni-B-Mo Coatings

Based on preliminary experiments on electroless Ni-B-Mo coatings, a series of pretreatment steps was established to ensure coating adhesion and quality. First, the substrate was sequentially ground using silicon carbide sandpapers (160#, 320#, 600#, and 1000#) and then polished with diamond suspension sprays (W2.5, W0.5) until a mirror-like surface was achieved. The polished samples were rinsed with deionized water and ethanol to remove surface contaminants. After air-drying, a hanging wire was attached to the specimen, which was then placed in anhydrous ethanol and ultrasonically cleaned for 10 min. Subsequently, the sample was immersed in a thermostatic alkaline solution at 60 °C (NaOH, 10 g/L; Na_2_CO_3_, 20–30 g/L; Na_3_PO_4_·12H_2_O, 50 g/L; Na_2_O·nSiO_2_, 5 g/L) for 15 min to remove grease. The specimen was then immersed in 30% hydrochloric acid for 1 min to activate the surface. After ultrasonic cleaning, alkaline cleaning, and acid activation, the sample was sequentially rinsed with hot and cold deionized water for 10 s each. Finally, the specimen was immediately immersed in the plating solution for deposition.

The electroless Ni-B-Mo plating solution was made up of nickel chloride hexahydrate as the main salt, sodium borohydride to aid reduction, ethylenediamine to help the formation of complexes, and sodium molybdate as the source of Mo. Lead nitrate was used as a stabilizer, while sodium hydroxide was employed as a pH regulator to maintain the pH above 13. We maintained the plating solution at 90 °C with constant stirring using a magnetic stirrer at a speed of 100 rpm. We conducted the deposition process for 1 to 4 h, starting with an initial solution volume of 500 mL. The specific composition and deposition conditions of the electroless Ni-B-Mo plating solution are summarized in Table 2.

We designed an orthogonal experiment with three factors, selecting the controllable parameters and their levels shown in Table 3. Since this study involved multiple factors with unequal numbers of levels, a mixed-level orthogonal array design was adopted to avoid excessive experimental combinations while maintaining statistical independence. Jiang and Yin [19] proposed a general fan construction method that can systematically generate MOAs with different strengths and level structures, which has been widely applied in material design and combinatorial optimization. The L_12_ (6 × 4 × 3) orthogonal array employed in this study conformed to their construction model. The experiment studied the influence of the deposition parameters on the structure and properties of the coating.

### 2.3. Ni-B-Mo Coating Characterization

1. The surface morphology, cross-sectional morphology, and elemental distribution of the coatings were characterized using a Japanese optical microscope (OLYMPUS-DSX510, Olympus Corporation, Yijingtong Optical Technology (Shanghai) Co., Shanghai, China) and a German scanning electron microscope (Zeiss Sigma 300, Carl Zeiss AG, Oberkochen, Germany). The coating thickness (µm) was measured, and the deposition rate (µm/h) was calculated.
2. Using the Dutch Empyrean X-ray diffractometer (Malvern Panalytical, Almelo, The Netherlands), the voltage was set to 40 kV and the current to 40 mA. Gr-Cu radiation was used, with a 2θ scanning range from 10° to 80°, for a qualitative analysis of the elemental composition of the coating surface.
3. The Japanese EDX-7200 energy-dispersive X-ray spectrometer (Shimadzu Corporation, Kyoto, Japan) was used to analyze the elemental content of the coating. Since the spectrometer could not detect elements lighter than oxygen, this experiment mainly used EDX spectrometry to determine the relative contents of Ni and Mo. The instrument had an error margin of ±0.3%, and the measurements were conducted under ambient air conditions at room temperature.
4. Using the Chinese HV-1000 microhardness tester (Shanghai Lianer Test Equipment Co., Ltd., Shanghai, China), a load of 100 g was applied along the depth direction of the coating with a dwell time of 10 s. The Vickers microhardness of the coating was measured at seven different points, and the average value was taken.
5. The Chinese HBE-3000A adhesion tester (Shanghai Lianer Test Equipment Co., Ltd.) was used to perform indentation testing, and the adhesion grade of the coating was qualitatively evaluated according to the VDI 3198 standard [20].
6. The American UMT-3 friction and wear tester (Bruker Nano Surfaces Division, Santa Barbara, CA, USA) was used to measure the tribological properties of the samples. Under room temperature and unlubricated conditions, the linear reciprocating mode was selected. The sample was cut into a thin 10 mm × 10 mm square sheet. The testing parameters were set as follows: a load of 10 N, a frequency of 2 Hz, a friction stroke of 5 mm, and a friction time of 30 min were used, and the friction pair used was a GCr15 steel ball with a diameter of 10 mm [21].

## 3. Results

### 3.1. The Effect of the Main Salt Concentration and Deposition Time on the Deposition Rate and Hardness of the Coating

#### 3.1.1. Deposition Rate of Ni-B-Mo Coatings

According to the orthogonal experimental design, the effect of each factor on the deposition rate was analyzed using the range values. $\bar{Ki}$ represented the sum of the reference performances at the ith level of each factor, and the corresponding average value was represented by Ki. R denoted the range [22]. In the orthogonal experiment, A1–A6, B1–B4, and C1–C3, respectively, represented the levels of the Na_2_MoO_4_ concentration (0.6–25.6 g/L), deposition time (1–4 h), and NiCl_2_ concentration (20–30 g/L). As the range value R increased, the impact of level changes on the reference parameter became more significant; conversely, when the range value R decreased, the impact of level changes on the reference parameter became less significant. Table 4 presents the deposition rate results for each group in the Ni-B-Mo chemical plating orthogonal experiment, while Table 5 shows the range analysis results for the deposition rate from the Ni-B-Mo chemical plating orthogonal experiment.

**Figure 1:**
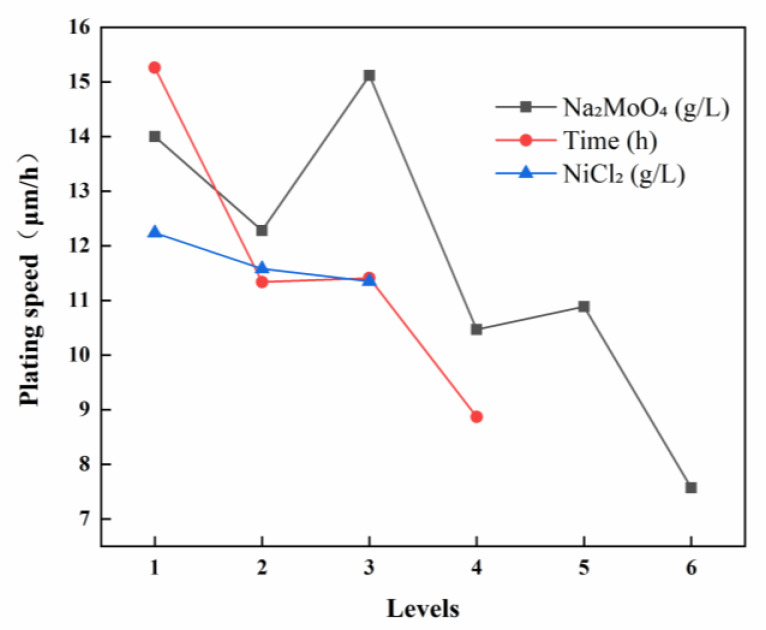
Ni-B-Mo coating deposition rate visual effect diagram.

Figure 1 illustrates the relationship between the deposition rate of the Ni-B-Mo coating and the sodium molybdate content and time.

**Figure 2:**
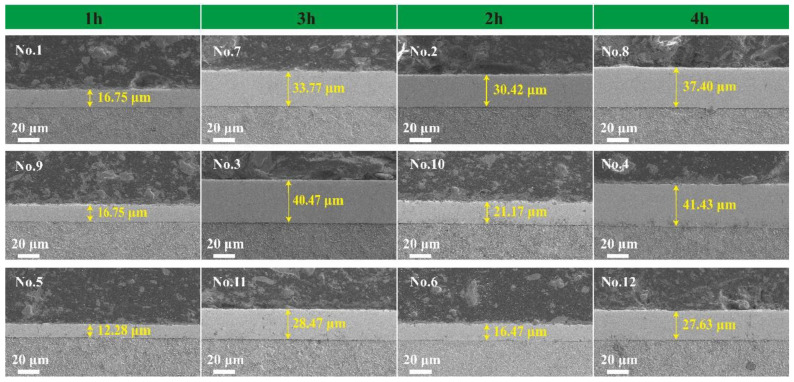
Cross-sectional morphology of Ni-B-Mo coatings: No. 1, No. 7: 0.6/20; No. 2, No. 8: 5.6/25; No. 3, No. 9: 10.6/30; No. 4, No. 10: 15.6/20; No. 5, No. 11: 20.6/25; No. 6, No. 12: 25.6/30.

Figure 2 presents the cross-sectional morphology images of 12 sets of Ni-B-Mo composite coatings analyzed using scanning electron microscopy. In experiments No. 1–No. 12, the different concentrations of Na_2_MoO_4_ and NiCl_2_ (g/L) were 0.6/20 (No. 1, No. 7); 5.6/25 (No. 2, No. 8); 10.6/30 (No. 3, No. 9); 15.6/20 (No. 4, No. 10); 20.6/25 (No. 5, No. 11); and 25.6/30 (No. 6, No. 12). Each pair of experiments (e.g., No. 1 and No. 7, No. 2 and No. 8) used the same concentration of Na_2_MoO_4_ and NiCl_2_. In the figure, the Na_2_MoO_4_ content increases by 10 g/L from the top to the bottom.

Orthogonal experimental analysis revealed that the concentration of sodium molybdate (Na_2_MoO_4_) had the most significant influence on the deposition rate of the coating, with a range value of R = 7.55. At a low Na_2_MoO_4_ concentration (0.6 g/L), the deposition rate was relatively low, resulting in a thin coating, as the insufficient molybdate content was unfavorable for coating formation. When the concentration increased to 10.6 g/L, the deposition rate reached a relatively high level, and the coating exhibited a uniform and dense structure. However, when the concentration was further increased to 25.6 g/L, the deposition rate decreased, and the coating surface became rough.

The plating time had the second most significant effect on the deposition rate, with a range value of R = 6.39. As the plating time increased, the overall coating thickness exhibited an increasing trend. With a short duration (1 h), the deposition rate was relatively high, allowing metal ions to rapidly deposit onto the substrate, resulting in fast and uniform coating formation. However, as the plating time was extended to 4 h, the deposition rate decreased, potentially due to the consumption of active components in the plating solution or diffusion limitations, which slowed down the deposition reaction.

In contrast, the concentration of nickel chloride (NiCl_2_) had a relatively minor effect on the deposition rate, with a range value of R = 0.89. Variations in the NiCl_2_ concentration within the range of 20–30 g/L had a minimal impact on the deposition rate, indicating that the Ni^2+^ ion concentration was not the primary determinant of the deposition rate.

Based on a comprehensive analysis, the sodium molybdate concentration was identified as the most critical factor influencing both the deposition rate and coating morphology, with an optimal concentration of 10.6 g/L. The plating time also had a significant impact on the deposition rate, with the optimal duration determined to be 1 h. The concentration of nickel chloride had a relatively minor influence, with variations within the range of 20–30 g/L having little effect on the deposition rate and morphology. The optimal experimental combination for achieving the highest deposition rate was determined to be A_3_B₁C₁ (Na_2_MoO_4_ = 10.6 g/L, t = 1 h, NiCl_2_ = 20 g/L).

#### 3.1.2. Hardness of Ni-B-Mo Coatings

Table 6 presents the hardness results for each group in the orthogonal experiment on Ni-B-Mo electroless plating, while Table 7 provides the range analysis of the hardness variations in the orthogonal experiment.

**Figure 3:**
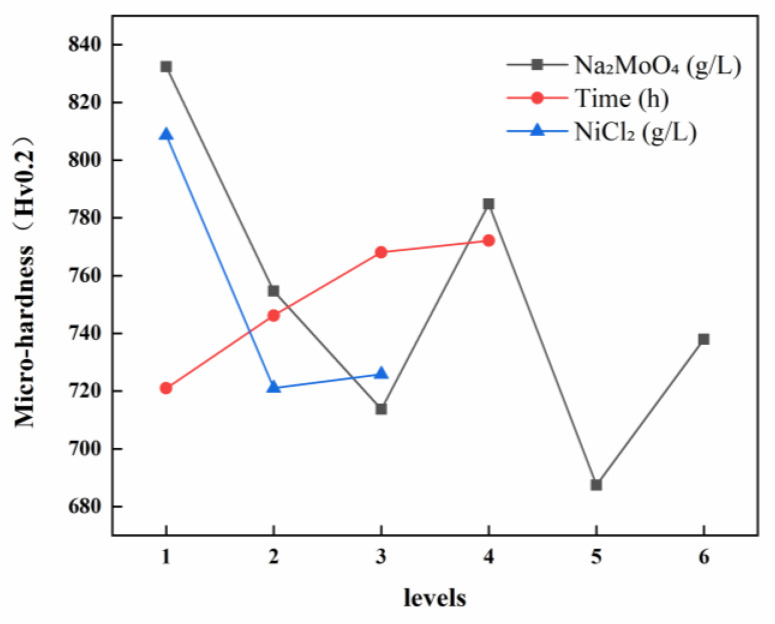
Visual representation of Ni-B-Mo coating microhardness.

Based on the visual representation of the microhardness of Ni-B-Mo coatings (Figure 3) and experimental data analysis, the primary factors influencing hardness were ranked as A (sodium molybdate concentration) > C (nickel chloride concentration) > B (plating time), with range values of 144.97, 87.54, and 51.15, respectively. At a low Na_2_MoO_4_ concentration (0.6 g/L), the coating exhibited relatively high hardness (881.00 HV), indicating an effective strengthening effect of the Ni-B-Mo structure. However, as the Na_2_MoO_4_ concentration increased to 10.6 g/L, the hardness decreased (to 727.76 HV), which may be attributed to the uneven distribution of Mo within the coating, thereby reducing its strengthening effect. When the concentration was further increased to 25.6 g/L, the hardness continued to decline (to 730.34 HV), possibly due to excessive Mo content leading to grain coarsening, which subsequently reduces the hardness of a coating.

The deposition time had a relatively minor effect on the hardness. At a short deposition duration (1 h), the coating hardness remained relatively low due to the insufficient thickness of the deposited layer, which prevented the formation of a fully developed Ni-B-Mo structure. As the deposition time increased to 3 h, the hardness rose rapidly, whereas a further extension to 4 h resulted in a slower increase, causing the maximum hardness of 814.08 HV to be reached. This indicates that a prolonged deposition time contributes to coating densification and grain refinement, thereby enhancing the hardness.

The effect of the NiCl_2_ concentration on the hardness was moderate. At a lower concentration (20 g/L), the coating exhibited the highest hardness (881.00 HV), indicating that an appropriate amount of Ni^2+^ facilitates the formation of a high-hardness Ni-B composite structure. As the NiCl_2_ concentration increased to 25 g/L, the hardness decreased (to 771.94 HV), likely due to an excessive Ni content, which would have led to an increased proportion of the softer Ni-based phase, thereby weakening the strengthening effect of the coating. At a higher concentration (30 g/L), the hardness declined further (to 699.61 HV), suggesting that an excessive Ni content suppresses the strengthening contributions of B and Mo within the coating.

A comprehensive analysis suggested that the optimal experimental combination was A_1_B_4_C_1_ (Na_2_MoO_4_ = 0.6 g/L, t = 4 h, NiCl_2_ = 20 g/L), under which conditions the coating achieved the highest hardness of 881.00 HV.

### 3.2. Ni-B-Mo Plating Bonding Strength

**Figure 4:**
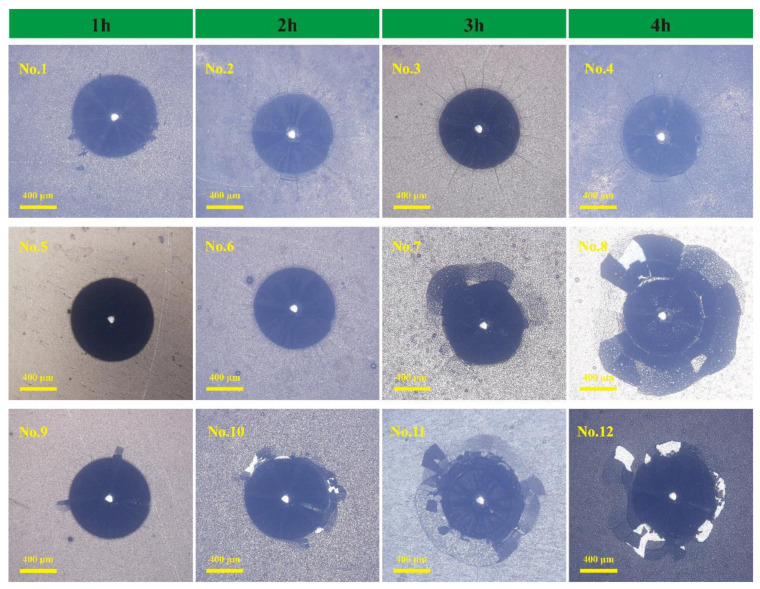
Ni-B-Mo plating bonding strength.

Figure 4 presents the adhesion indentation images of Ni-B-Mo coatings for twelve different combinations of the sodium molybdate concentration, nickel chloride concentration, and plating time (magnification: 139×).

At low sodium molybdate concentrations (0.6 g/L, No. 1), the coating exhibited strong adhesion, with only minor annular cracks and a few short radial cracks around the indentation. As the sodium molybdate concentration increased (5.6–15.6 g/L, No. 2, No. 4, No. 8, and No. 10), the adhesion strength of the coating remained moderate, with cracks of a moderate length and some radial cracks penetrating the coating, displaying a certain degree of brittleness but without severe delamination. However, when the sodium molybdate concentration was high (25.6 g/L, No. 11 and No. 12), there were many more radial cracks and large areas where the coating separated. This evidence indicates that excessive Mo doping leads to grain coarsening and structural defects, which in turn reduce the adhesion strength of a coating.

The concentration of nickel chloride also had a significant impact on the adhesion strength of the coating. Under low nickel chloride concentrations (20 g/L), the coating exhibited strong adhesion, with fewer cracks around the indentation and excellent density, as seen in No. 1, No. 7, etc. At moderate nickel chloride concentrations (25 g/L), the crack length around the indentation slightly increased, but no delamination was observed, with the adhesion strength remaining at a moderate level, as seen in No. 2, No. 5, etc. This could potentially be attributed to the rapid deposition rate leading to a concentration of stress. At high nickel chloride concentrations (30 g/L), the rapid deposition rate led to grain coarsening, increased porosity, and coating embrittlement. The adhesion strength consequently declined dramatically, resulting in large-scale delamination and noticeable radial cracks, as demonstrated in Nos. 3 and 9.

The effect of the plating time on the adhesion strength was as follows: For short plating times (1–2 h), the coating thickness was moderate, the residual stress was low, there were fewer cracks around the indentation, and no significant delamination occurred, resulting in strong adhesion (e.g., No. 1 and No. 6). As the plating time was extended to 3 h, the coating thickness further increased, with some areas showing radial cracks, indicating the concentration of residual stress and an increased tendency for crack propagation (e.g., No. 3 and No. 11). Under long plating times (4 h), a significant amount of internal stress occurred, with grain boundary weakening leading to noticeable delamination. Consequently, the adhesion strength dropped significantly (e.g., No. 8 and No. 12).

### 3.3. Ni-B-Mo Surface Morphology

**Figure 5:**
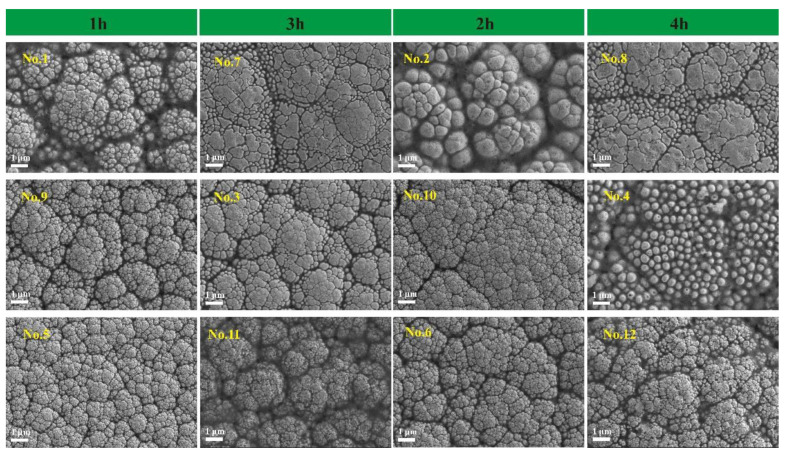
Ni-B-Mo surface morphology: No. 1, No. 7: 0.6/20; No. 2, No. 8: 5.6/25; No. 3, No. 9: 10.6/30; No. 4, No. 10: 15.6/20; No. 5, No. 11: 20.6/25; No. 6, No. 12: 25.6/30.

Figure 5 presents the surface scanning electron microscope images of the Ni-B-Mo coatings prepared under different conditions. In experiments No. 1 to No. 12, the different concentrations of Na_2_MoO_4_ and NiCl_2_ (g/L) were 0.6/20 (No. 1, No. 7), 5.6/25 (No. 2, No. 8), 10.6/30 (No. 3, No. 9), 15.6/20 (No. 4, No. 10), 20.6/25 (No. 5, No. 11), and 25.6/30 (No. 6, No. 12), with each pair of codes corresponding to the same concentrations of Na_2_MoO_4_ and NiCl_2_ (e.g., No. 1 and No. 7 both correspond to 0.6/20). The surface of the coatings exhibited a particulate structure, which is characteristic of the columnar growth typical of Ni-B-based electroless plating [23,24,25]. Based on the size of the columnar particles and the surface morphology, the 12 coating groups were classified as having a blackberry shape (No. 1, No. 2), cracked mud appearance (No. 3, No. 7, No. 8), fish egg shape (No. 4), cauliflower shape (No. 5, No. 6, No. 9, No. 10), or broccoli shape (No. 11, No. 12).

**Figure 6:**
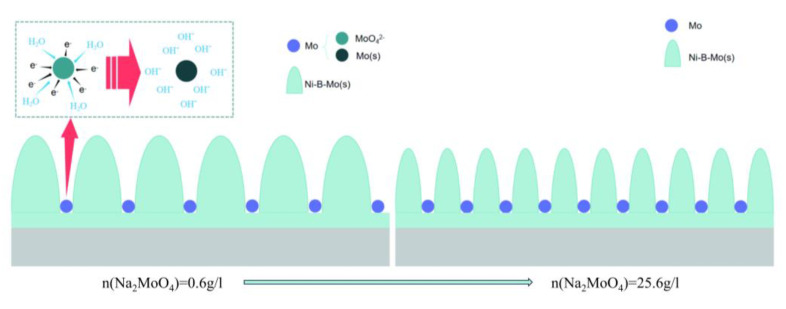
Schematic diagram of effect of sodium molybdate content on columnar growth in Ni-B-Mo coating.

Under the same plating time, the Na_2_MoO_4_ concentration increased by 10 g/L (from the top to the bottom in the figure), leading to a gradual reduction in the size of the columnar particles. This was due to the increased Na_2_MoO_4_ concentration, which caused the solution to become more supersaturated, accelerating the bonding rate and increasing the number of solid metal compounds, reducing aggregation phenomena and thus resulting in smaller particle growth. This situation was mainly caused by the effect of metal co-deposition. When the Na_2_MoO_4_ concentration is low, it accelerates the decomposition and oxidation of borohydride ions, which enhances the reduction of nickel ions to some extent, enabling the co-deposition of a ternary Ni-B-Mo alloy. This plays a catalytic role similar to that of chemicals used to accelerate deposition reactions. When the Na_2_MoO_4_ concentration is high, it acts as an inhibitor or stabilizer, similarly to chemicals used to slow down deposition reactions. Free molybdenum adsorbed on the activated metal surface or the newly formed coating surface blocks the bonding of the deposits with the metal or coating. As the Na_2_MoO_4_ concentration increased in the plating solution, more free molybdenum was generated on the substrate, resulting in thinner columnar structures, making the morphology of the second and third rows denser with smaller particle sizes compared to the first row, as shown in Figure 6.

By comparing columns 1 and 2 and columns 3 and 4 (in Figure 5), it can be observed that with an increasing time, under conditions of low (0.6, 5.6 g/L) and high (20.6, 25.6 g/L) Na_2_MoO_4_ concentrations, the size of the columnar particles gradually decreased, as seen in No. 1→No. 7 and No. 5→No. 11. However, under medium Na_2_MoO_4_ concentrations (15.6, 20.6 g/L), the columnar particle size gradually increased, as seen in No. 9→No. 3 and No. 10→No. 4. Under the same plating solution conditions, increasing the plating time resulted in the formation of more pores. Specifically, under higher Na_2_MoO_4_ concentrations, when the plating time reached 3–4 h, the surface took on a broccoli-like appearance, with fine and loose particles, as seen in No. 11 and No. 12 in Figure 5. In comparison, under low Na_2_MoO_4_ concentrations (No. 1, No. 7, No. 2, No. 8), the surface uniformity decreased, and localized bonding resulting in the formation of recessed blocks occurred, with a significant amount of single-columnar growth still present, as can be seen in Figure 5. This is because, as the plating time increased, the coating thickness gradually increased, reducing the coating’s ability to adsorb deposited particles.

Under low NiCl_2_ concentrations (20 g/L), the particles were large, grew slowly, and had high surface roughness and poor uniformity, forming a blackberry-like or irregular columnar structure (e.g., No. 1, No. 4, No. 7, No. 10). At moderate NiCl_2_ concentrations (25 g/L), the coating particles were of a medium size and densely arranged, forming an even cauliflower-like structure (e.g., No. 6, No. 9). At high NiCl_2_ concentrations (30 g/L), the coating particles became further refined, making the coating denser, with a surface tending towards a broccoli shape (e.g., No. 11).

In summary, an appropriate amount of Na_2_MoO_4_ (10.6–15.6 g/L), a moderate NiCl_2_ concentration (25 g/L), and an optimal plating time (2–3 h) can produce Ni-B-Mo coatings with uniform particles, a high density, and a good surface quality.

### 3.4. Component Characterization and Analysis

#### 3.4.1. XRD Characterization and Analysis

**Figure 7:**
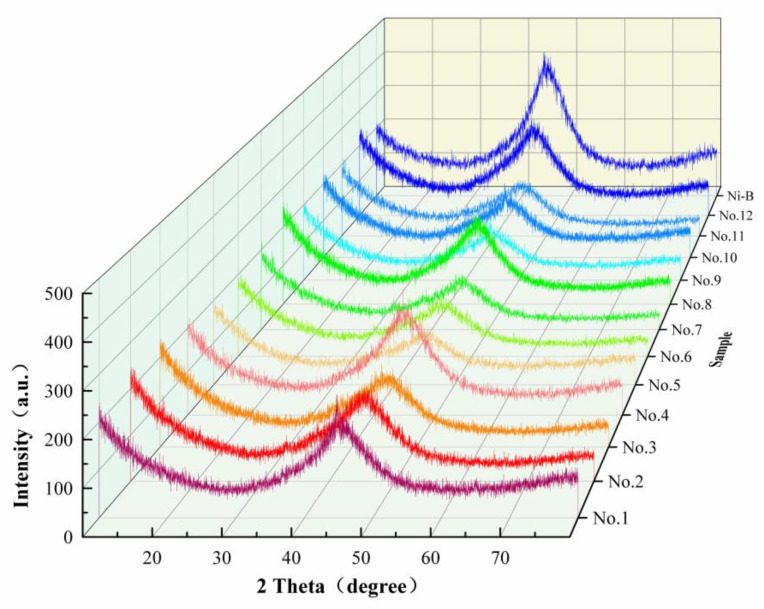
XRD diffractograms of Ni-B-Mo and Ni-B coatings.

Figure 7 displays the X-ray diffraction patterns of Ni-B-Mo coatings and Ni-B coatings for twelve different combinations of the Na_2_MoO_4_ concentration, NiCl_2_ concentration, and deposition time. The XRD patterns revealed that the structure of Ni-B-Mo coatings evolved from crystalline to amorphous as the Na_2_MoO_4_ concentration and deposition time changed. Under a low Na_2_MoO_4_ concentration (e.g., 0.6 g/L, No. 1), the XRD pattern showed a lower main peak intensity and broader peak width, with a pronounced broad peak in the low-angle region, confirming the coexistence of crystalline and amorphous phases, with a higher proportion of the amorphous phase [26]. This was attributed to the low concentration of Mo, which limited the lattice stability, and the short deposition time, which resulted in limited crystallinity. With the same bath concentration, as the deposition time increased (e.g., No. 7), the main peak intensity further weakened and broadened, while the broad amorphous peak increased in strength, indicating that prolonged deposition enhances lattice distortion and the proportion of the amorphous phase.

As the concentration of Na_2_MoO_4_ went up (for example, from 5.6 to 15.6 g/L in samples No. 2, No. 4, No. 8, and No. 10), the main peak became wider and weaker, while the broad peak became much stronger, showing that Mo doping started to affect how nickel formed, leading to both crystalline and amorphous phases being present. At intermediate concentrations (e.g., No. 6), the ratio of crystalline to amorphous phases in the coating reached a more balanced state, where both crystalline diffraction peaks (e.g., the (111) peak of Ni) and a distinct broad amorphous peak were observable. Under high Na_2_MoO_4_ concentrations (e.g., 20.6–25.6 g/L, No. 5 and No. 11), the main peak significantly weakened or even disappeared, while the broad peak strengthened significantly, indicating that the coating structure gradually transitioned to complete amorphization, with the No. 11 sample being close to complete amorphization.

A Ni-B coating is a typical amorphous structure, where disorder in the atomic arrangement leads to the appearance of broad peaks, and the degree of boron segregation in the coating determines its crystallinity [27]. By comparing the Ni-B-Mo coating with a Ni-B coating, it was found that the presence of Mo largely suppressed the nucleation of nickel ions, leading to the transition of the Ni-B-Mo coating from a crystalline–amorphous coexistence to a completely amorphous structure.

Therefore, at low sodium molybdate concentrations (0.6 g/L), the coating predominantly exhibited a crystalline–amorphous coexistence. As the concentration increased, Mo doping disrupted the crystalline phase of Ni, promoting the coating’s transition toward an amorphous state. An increase in the nickel chloride concentration (20 g/L–30 g/L) also promoted the formation of an amorphous structure, with Mo’s effect becoming more pronounced under higher concentrations of nickel chloride. When the plating time increased from 1 to 4 h, exacerbating lattice distortion, it increased the proportion of the amorphous phase and ultimately led to complete amorphization.

#### 3.4.2. SEM Characterization and Analysis

**Figure 8:**
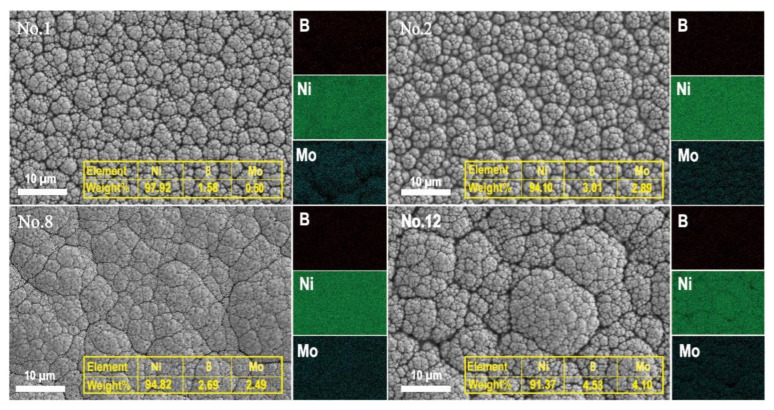
Surface microstructure and EDS mapping image of Ni-B-Mo coating: No. 1 (0.6 g/L Na_2_MoO_4_, 1 h, 20 g/L NiCl_2_); No. 2 (5.6 g/L Na_2_MoO_4_, 2 h, 25 g/L NiCl_2_); No. 8 (5.6 g/L Na_2_MoO_4_, 4 h, 25 g/L NiCl_2_); No. 12 (25.6 g/L Na_2_MoO_4_, 4 h, 30 g/L NiCl_2_).

Further analysis was conducted on the element mapping and microstructure of the coatings from groups 1, 2, 8, and 12. No. 1 (0.6 g/L Na_2_MoO_4_, 1 h, 20 g/L NiCl_2_) represented the coating formed under a low sodium molybdate concentration, short deposition time, and low nickel chloride concentration; No. 2 (5.6 g/L Na_2_MoO_4_, 2 h, 25 g/L NiCl_2_) represented the coating formed under a medium sodium molybdate concentration and nickel chloride concentration and moderate deposition time; No. 8 (5.6 g/L Na_2_MoO_4_, 4 h, 25 g/L NiCl_2_) represented the coating formed under an extended deposition time and medium sodium molybdate concentration and nickel chloride concentration; No. 12 (25.6 g/L Na_2_MoO_4_, 4 h, 30 g/L NiCl_2_) represented the coating formed under a high sodium molybdate concentration, long deposition time, and high nickel chloride concentration. Figure 8 shows the distribution of key elements under different bath concentrations and plating times, with red, green, and light blue representing the boron, nickel, and molybdenum in the coating, respectively.

Under low-concentration and short deposition time conditions (No. 1, 0.6 g/L Na_2_MoO_4_, 1 h), the coating primarily exhibited a crystalline phase. Elemental mapping showed a uniform distribution of Ni, with relatively low and sparsely distributed B and Mo content. The particles were fine and evenly dispersed, resulting in a dense coating structure with a low degree of amorphization. Under medium-concentration and intermediate deposition time conditions (No. 2, 5.6 g/L Na_2_MoO_4_, 2 h), the particle size increased significantly compared to that of No. 1. Ni remained uniformly distributed, while the B and Mo content increased. The coating structure progressively transitioned toward amorphization while maintaining a good density. The interparticle bonding was relatively tight, leading to a more uniform microstructure. When the deposition time was further extended (No. 8, 5.6 g/L Na_2_MoO_4_, 4 h), the particle size slightly decreased compared to that of No. 2, but the boundaries became indistinct, indicating increased structural densification. The Mo content slightly decreased, while the degree of amorphization further increased. Under high-concentration and prolonged deposition time conditions (No. 12, 25.6 g/L Na_2_MoO_4_, 4 h), the coating particles became significantly larger and more loosely packed. The B and Mo content increased substantially, leading to weaker interparticle bonding and a notable decrease in the coating density. Although Ni remained evenly distributed, Mo doping interfered with the crystallization process, resulting in an almost completely amorphous structure and a significant increase in the surface roughness.

Further investigation showed that the amount of Na_2_MoO_4_ and how long it was applied for greatly affected how the coating was formed and its properties. A low amount and short application time (0.6 g/L Na_2_MoO_4_, 1 h) helped create a solid, crystal-like structure, while a high amount and longer application time (25.6 g/L Na_2_MoO_4_, 4 h) led to a more disordered structure. Using a low amount of Na_2_MoO_4_ for a short time (0.6 g/L Na_2_MoO_4_, 1 h) helped create a solid, crystal-like structure, while using a high amount for a longer time (25.6 g/L Na_2_MoO_4_, 4 h) led to a more disordered structure. Mo doping played a key role in the amorphization process by inhibiting the nucleation of Ni crystals.

### 3.5. Friction and Wear Analysis and Determination of Optimal Ni-B-Mo Coating Parameters

#### 3.5.1. Preliminary Selection of Optimal Ni-B-Mo Coating

From the perspective of coating adhesion, experimental groups with poor adhesion were first excluded. Consequently, among the 12 experimental groups, 8 groups remained as viable candidates: No. 1, No. 2, No. 4, No. 5, No. 6, No. 8, No. 9, and No. 10. Further analysis of the surface morphology of the Ni-B-Mo coating samples revealed that the No. 4 sample exhibited excessive porosity, leading to its exclusion. Based on an analysis of the deposition rate, the Ni-B-Mo coating demonstrated superior deposition performance when the sodium molybdate concentration was within the range of 5.6–10.6 g/L and the deposition time was between 1.5 and 2.5 h, exhibiting significantly higher deposition rates than those of the other groups. Consequently, groups No. 1, No. 5, No. 6, and No. 10 were excluded. From the perspective of microhardness, a higher hardness value is more suitable for the actual working conditions of GCr15 steel. The microhardness of the No. 9 Ni-B-Mo coating was 699.61 HV0.2, whereas the microhardness values of the No. 2 and No. 8 coatings were 737.49 HV0.2 and 771.94 HV0.2, respectively, both exceeding 700 HV0.2. Therefore, groups No. 2 and No. 8 were selected for the friction and wear tests. To compare the effects of the sodium molybdate concentration, deposition time, and nickel chloride concentration on the wear resistance of the coating, group No. 12 was selected as a time control group. Ultimately, Ni-B-Mo coating samples from groups No. 2, No. 8, and No. 12 in the orthogonal experiment were selected for friction and wear testing alongside the GCr15 substrate.

#### 3.5.2. Tribological Behavior Analysis of Coating Samples

**Figure 9:**
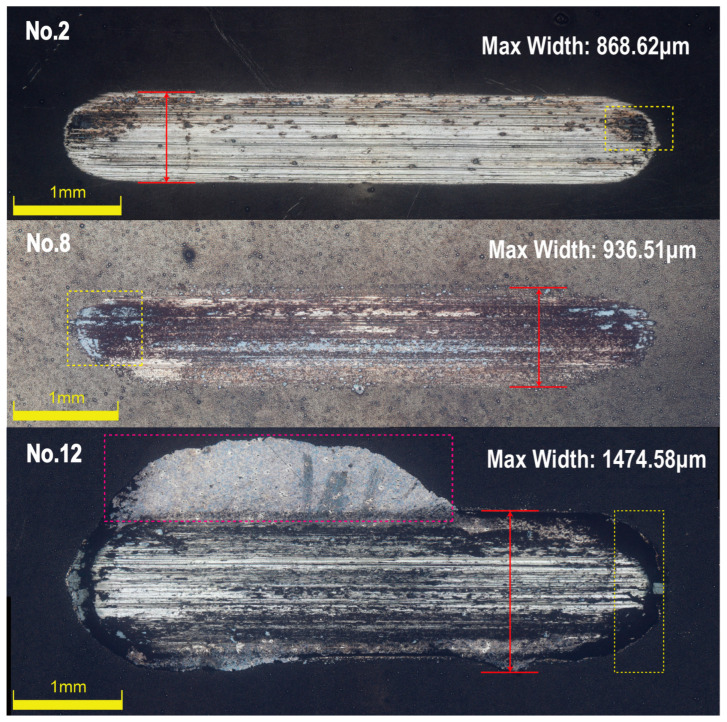
Wear morphology of Ni-B-Mo coatings: No. 2 (5.6 g/L Na_2_MoO_4_, 2 h, 25 g/L NiCl_2_); No. 8 (5.6 g/L Na_2_MoO_4_, 4 h, 25 g/L NiCl_2_); No. 12 (25.6 g/L Na_2_MoO_4_, 4 h, 30 g/L NiCl_2_).

Figure 9 illustrates the wear track morphology of the coatings under different deposition conditions. No. 2 (5.6 g/L Na_2_MoO_4_, 2 h, 25 g/L NiCl_2_) represented the coating deposited under moderate sodium molybdate and nickel chloride concentrations with an optimal deposition time. No. 8 (5.6 g/L Na_2_MoO_4_, 4 h, 25 g/L NiCl_2_) corresponded to the coating deposited under an extended deposition time but with the same moderate sodium molybdate concentration. No. 12 (25.6 g/L Na_2_MoO_4_, 4 h, 30 g/L NiCl_2_) represented the coating formed under a high sodium molybdate concentration, prolonged deposition time, and high nickel chloride concentration.

The wear scars on the GCr15 substrate exhibited a comet-like pattern, indicating poor wear resistance [28]. In contrast, the wear scars on the Ni-B-Mo coatings were elliptical, which aligned with the theoretical wear pattern observed in reciprocating friction tests. In the reciprocating friction test, sample No. 2 exhibited only a small amount of wear debris that accumulated at both ends of the wear scar, appearing dark gray. Due to the short deposition time, as shown in Figure 2, the coating thickness of sample No. 2 was relatively thin, with a lower Mo content. The smaller wear scar resulted in less frictional heating, leading to minimal oxidation. Consequently, the wear debris primarily consisted of metallic fragments from the Ni-B matrix. As can be seen in Figure 4, samples No. 8 and No. 12 exhibited weaker coating adhesion, generating a large amount of wear debris that spread from the wear scar to both ends. As the deposition time increased, the Mo content in sample No. 8 rose. Localized high temperatures during the application of friction accelerated the oxidation of Mo, forming MoO_3_. The wear debris consisted of larger particles that accumulated on both sides, appearing white under optical reflection. Sample No. 12, deposited for 4 h under a high Na_2_MoO_4_ concentration, exhibited an increased Mo content, leading to the formation of additional oxides such as MoO_2_ during the application of friction. This resulted in increased coating brittleness, with partial delamination forming black carbide debris, giving the wear debris a black appearance. The maximum wear scar widths for the GCr15 substrate and the three coating samples were 1310 μm, 868.62 μm, 936.51 μm, and 1474.58 μm, respectively. A smaller wear scar width indicates better wear resistance. Overall, the optimal combination for wear resistance was 5.6 g/L Na_2_MoO_4_, a 2 h deposition time, and 25 g/L NiCl_2_ (sample No. 2). In contrast, high Na_2_MoO_4_ (25.6 g/L) and NiCl_2_ (30 g/L) concentrations (sample No. 12) reduced the coating’s wear resistance, resulting in severe wear.

**Figure 10:**
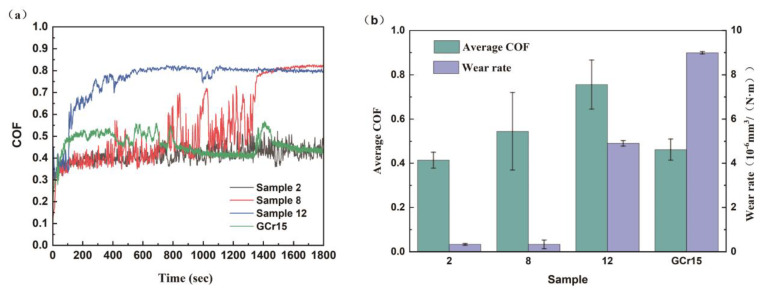
(**a**) Friction coefficient curve; (**b**) average friction coefficient and wear rate.

Figure 10a presents the friction coefficient curves of the GCr15 steel substrate and three coating samples with varying Na_2_MoO_4_ concentrations and deposition times. Under identical dry friction conditions, the COF of the sample with 5.6 g/L Na_2_MoO_4_ and a deposition time of 4 h rapidly increased from an initial value of 0.4 to 0.8, fluctuating significantly between 0.4 and 0.8. This indicates that the columnar growth at the initial deposition stage led to a cauliflower-like surface morphology, ultimately reducing the COF of the as-deposited coating by decreasing the actual contact area between the surfaces [29]. During the initial stage, the coating surface remained smooth. However, as the application of friction progressed, insufficient coating adhesion led to particle detachment, which was the primary cause of the severe COF fluctuations. For the sample with 25.6 g/L Na_2_MoO_4_ deposited for 4 h, the COF remained consistently high. This was attributed to the high Mo concentration, which resulted in coarse-grained coating surfaces with cracks. The abrasive debris generated at the beginning of the sliding test entered the wear track, further increasing the COF. The presence of Na_2_MoO_4_ promoted the formation of a rough, coarse-grained surface structure, which, under high stress, developed cracks. The coarse-grained surface morphology was responsible for the high surface roughness, ultimately leading to the increased COF of the coating. According to the classical friction theory proposed by Bowden and Tabor [30], the friction coefficient (COF) is given by

(1) $$\mu =\left(\tau \cdot A_{r}\right)/F_{N}$$

where $F_{N}$ is the normal force, $A_{r}$ is the real contact area at the friction interface, and τ is the shear strength of the material. Under identical friction conditions, the sample with 5.6 g/L Na_2_MoO_4_ and a deposition time of 2 h exhibited the highest hardness. This resulted in a lower real contact area, $A_{r},$ with GCr15 steel during the application of friction, leading to the lowest COF with minimal fluctuations.

Figure 10b presents the average friction coefficient and wear rate of the GCr15 steel substrate and Ni-B-Mo coatings deposited under three different experimental conditions. Under the optimized deposition conditions of 5.6 g/L Na_2_MoO_4_ and a deposition time of 2 h, the lowest wear rate of 0.3024 × 10^−6^ mm^3^/(N·m) was achieved, representing a reduction of 96.26% and 93.14% compared to that of the GCr15 steel substrate and the coating deposited at 25.6 g/L Na_2_MoO_4_ for 4 h, respectively. Moreover, this condition resulted in the lowest average wear rate, further confirming its superior tribological performance.

**Figure 11:**
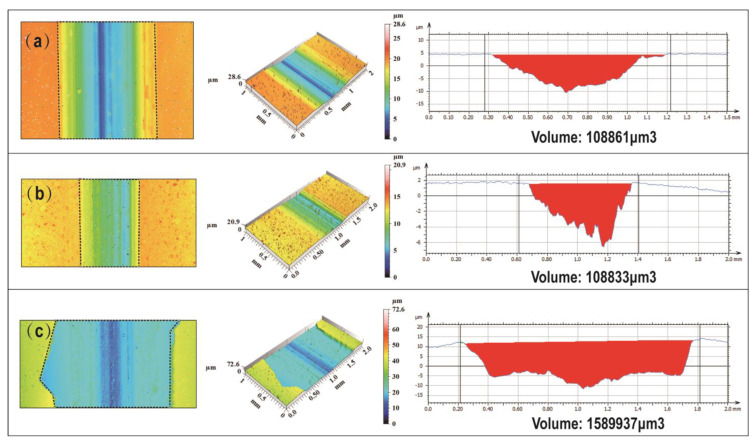
Three-dimensional morphology of wear tracks: (**a**) 5.6 g/L Na_2_MoO_4_, 2 h, 25 g/L NiCl_2_, (**b**) 5.6 g/L Na_2_MoO_4_, 4 h, 25 g/L NiCl_2_, (**c**) 25.6 g/L Na_2_MoO_4_, 4 h, 30 g/L NiCl_2_.

A laser confocal microscope was used to further examine the localized wear volume, surface roughness, and cross-sectional morphology of the coatings under three different experimental conditions. The wear volumes of sample 2, sample 8, and sample 12 were 108,861 μm^3^, 108,833 μm^3^, and 1,589,937 μm^3^, respectively. The cross-section of the wear track in sample 2 exhibited a regular and symmetric groove structure, indicating a uniform distribution of contact stress during the wear process. This uniformity ensured a consistent wear rate and depth across the coating. The wear track cross-section of sample 8 exhibited an irregular groove structure with significant surface undulations and a rough bottom. This suggests that a prolonged deposition time increases the coating thickness but also leads to residual stress accumulation during the deposition process. Under frictional loading, the presence of stress concentration zones promotes crack propagation, resulting in localized micro-spalling. The wear track cross-section of sample 12 was irregular and asymmetric, with a significantly wider and deeper groove, indicating poor wear resistance. Two primary factors contributed to this phenomenon: (1) An excessive Na_2_MoO_4_ concentration accelerated Mo deposition. As can be observed in Figure 11 (SEM surface morphology), the coating exhibited an uneven and coarse crystalline structure. Under frictional loading, the local stress concentration occurred more readily, promoting crack propagation and coating delamination. (2) An excessive Na_2_MoO_4_ concentration led to a transition from a fine-grained to coarse-grained coating structure. This weakened the grain boundaries, making the coating more brittle and reducing its impact resistance.

#### 3.5.3. Optimization of Ni-B-Mo Coating Process Parameters for GCr15 Steel

Based on a comprehensive analysis of the coating deposition rate, microhardness, and wear resistance, the optimal process conditions for a Ni-B-Mo coating on GCr15 steel were determined to be a Na_2_MoO_4_ concentration of 5.6 g/L, a NiCl_2_ concentration of 25 g/L, and a plating duration of 2 h. Under these conditions, the coating exhibited excellent adhesion, a high deposition rate, superior microhardness, and enhanced wear resistance. The optimal process parameters are summarized in Table 8.

### 3.6. Best Ni-B-Mo Coating Heat Treatment Analysis

Moderate heat treatment can enhance the performance of nickel-based alloy coatings, making it crucial to study the impact of heat treatment on the coating [31]. Numerous studies have shown that the optimal heat treatment temperature for Ni-B-based binary and multi-component alloy coatings is typically around 400 °C. When the heat treatment temperature of Ni-B coatings exceeds 350 °C, the coating structure transitions from an amorphous to a crystalline state, resulting in improved bonding strength, hardness, wear resistance, and other properties [11,32,33].

Therefore, in this study, an SX2–4-12TP box-type resistance furnace was selected to carry out heat treatment on the optimal Ni-B-Mo coating on GCr15 steel samples. The coating was heated at a rate of 10 °C/min for 40 min, reaching 400 °C and holding for 1 h, followed by slowly cooling to room temperature through furnace cooling.

#### 3.6.1. Surface Morphology Results and Analysis

**Figure 12:**
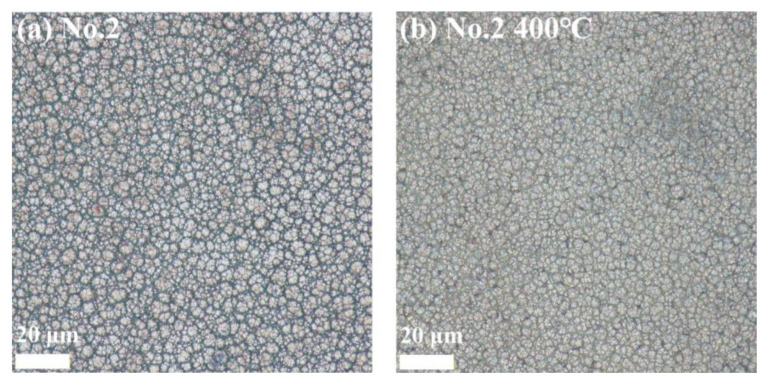
Surface morphology of the best Ni-B-Mo coating on GCr15 steel: (**a**) Ni-B-Mo coating before heat treatment and (**b**) after heat treatment at 400 °C for 1 h.

**Figure 13:**
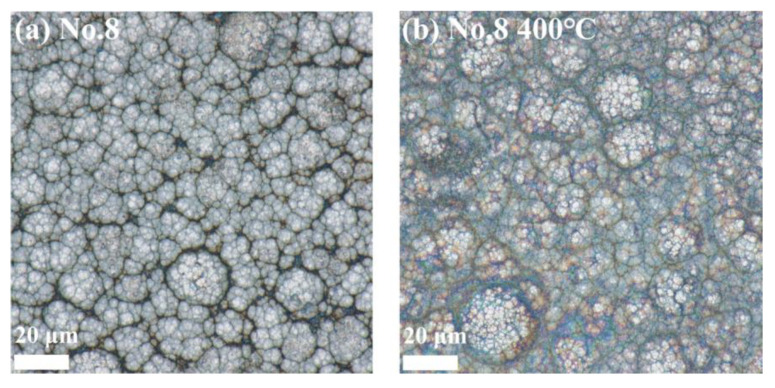
Surface morphology of Ni-B-Mo coating (No. 8: Na_2_MoO_4_ at 5.6 g/L, NiCl_2_ at 25 g/L, t of 4 h): (**a**) Ni-B-Mo coating before heat treatment and (**b**) after heat treatment at 400 °C for 1 h.

**Figure 14:**
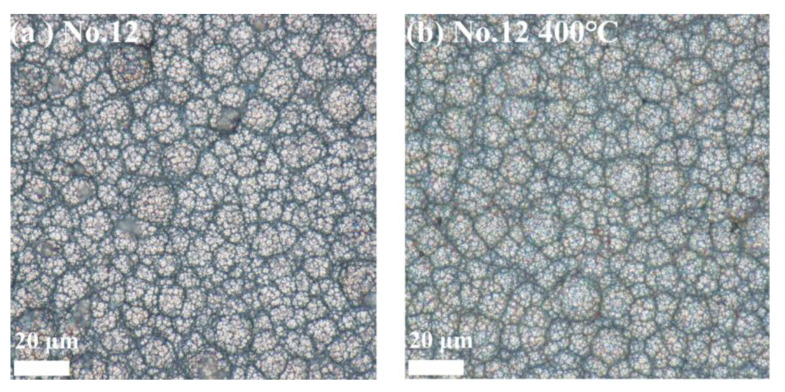
Surface morphology of Ni-B-Mo coating (No. 12: Na_2_MoO_4_ at 25.6 g/L, NiCl_2_ at 30 g/L, t of 4 h): (**a**) Ni-B-Mo coating before heat treatment and (**b**) after heat treatment at 400 °C for 1 h.

Figure 12, Figure 13 and Figure 14 show the surface morphology of the Ni-B-Mo coatings before and after heat treatment at 400 °C for 1 h. The optimal Ni-B-Mo coating No. 2 (Figure 12) exhibited a fine and evenly distributed spherical particle structure after deposition, while after heat treatment at 400 °C, the boundaries between the particles became blurred, possibly due to grain growth, element diffusion, and an increased coating density. The No. 8 coating (Figure 13), formed under a lower Na_2_MoO_4_ concentration (5.6 g/L) and a longer deposition time (4 h), showed a noticeable increase in the particle size, with clear boundaries. After heat treatment at 400 °C, darker regions appeared, possibly due to compound oxidation, and although the particle boundaries remained clear, the particle size increased in certain areas. The No. 12 coating (Figure 14), formed under a higher Na_2_MoO_4_ concentration (25.6 g/L) and a longer deposition time (4 h), exhibited a denser particle arrangement, with grain sizes similar to those of No. 2. After heat treatment at 400 °C, the surface morphology changed little, and compared to No. 8, the particle boundaries remained clearer. Overall, the surface morphology of the coatings was minimally affected by heat treatment, with only noticeable changes in the particle boundary definition and color deepening.

#### 3.6.2. XRD Spectrum Results and Analysis

**Figure 15:**
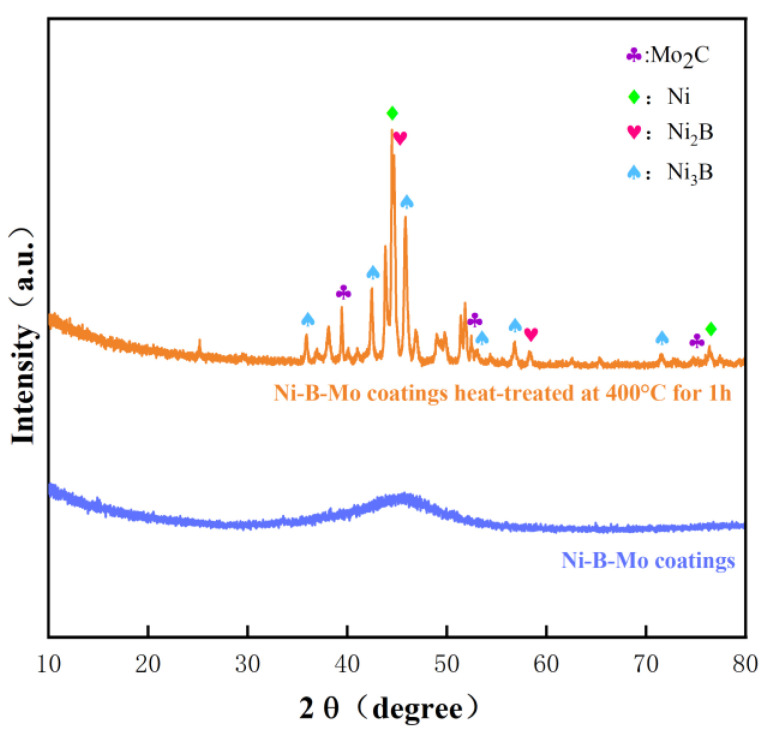
XRD patterns of Ni-B-Mo coating before and after heat treatment at 400 °C for 1 h.

Figure 15 shows the XRD spectra of the optimal Ni-B-Mo coating before and after heat treatment at 400 °C for 1 h. The figure shows that the as-deposited Ni-B-Mo coating had an amorphous structure, which is indicated by the wide peak at around 45° in the XRD spectrum, showing that it was not crystalline. Theoretically, a broad hump should have appeared at 2θ = 45°, but the XRD results showed a sharp peak at 2θ = 44.7°. The main reason for this was that during the heat treatment, the B and Mo elements in the coating formed compounds, which caused the small proportion of the amorphous phase to slowly change to a nanocrystalline state. After holding the heat treatment for 1 h, the number of nanocrystals increased, and the intensity of the characteristic peaks also strengthened. However, XRD analysis revealed that the coating clearly transformed from an amorphous to a crystalline state when it was heat-treated at 400 °C or higher. Nickel crystalline phases (JCPDS No. 70-1849) appeared at 2θ = 44.4° and 76.3°, indicating the crystallization of the nickel phase. Furthermore, the orange spectrum in the figure shows the presence of nickel borides and molybdenum carbide phases after heat treatment at 400 °C, indicating the formation of Ni_2_B, Ni_3_B, and Mo_2_C phases after 1 h of heat treatment at 400 °C. Specifically, the Ni_2_B phase (JCPDS No. 75-1064) was detected at 2θ = 44.7° and 58.5°; the Ni_3_B phase (JCPDS No. 82-1699) at 2θ = 36.9°, 42.4°, 46.0°, 53.0°, 56.8°, and 71.4°; and the Mo_2_C phase (JCPDS No. 31-0871) at 2θ = 39.4°, 52.2°, and 74.7°. It can be concluded that these phases existed as solid solutions in the Ni-B-Mo coating.

#### 3.6.3. Bonding Strength Results and Analysis

**Figure 16:**
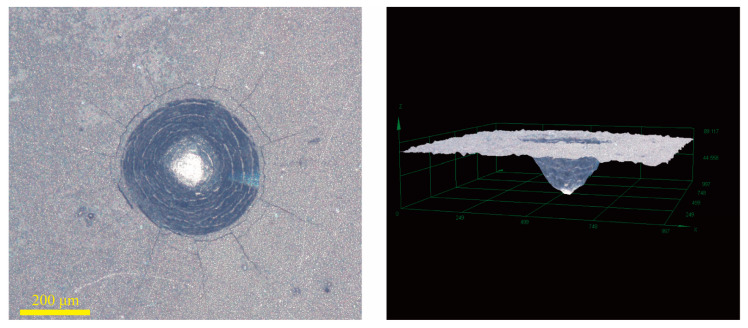
Two-dimensional and three-dimensional graphs showing the bonding strength of the best Ni-B-Mo coating for GCr15 steel.

**Figure 17:**
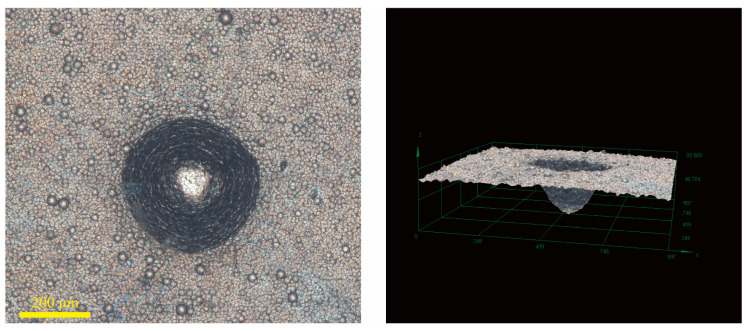
Two-dimensional and three-dimensional graphs showing the bonding strength of the Ni-B-Mo coating before and after heat treatment at 400 °C for 1 h (No. 8: Na_2_MoO_4_ = 5.6 g/L, NiCl_2_ = 25 g/L, t = 4 h).

**Figure 18:**
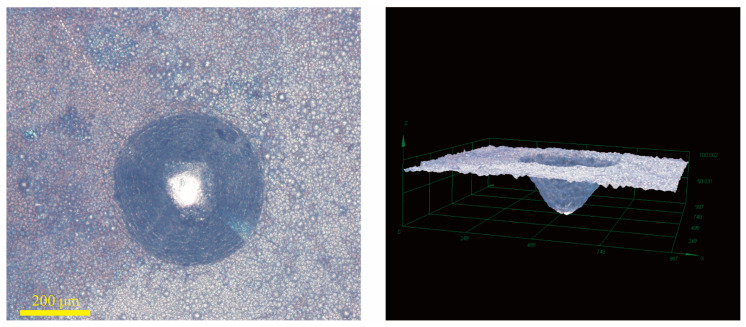
Two-dimensional and three-dimensional graphs showing the bonding strength of the Ni-B-Mo coating before and after heat treatment at 400 °C for 1 h (No. 12: Na_2_MoO_4_ = 25.6 g/L, NiCl_2_ = 30 g/L, t = 4 h).

Figure 16, Figure 17 and Figure 18 show the bonding strength of the Ni-B-Mo coating on GCr15 steel under different process conditions. The left side displays 2D surface morphology images, illustrating the crack propagation, while the right side presents 3D morphology images, reflecting the delamination extent of the coating. Figure 16 shows the optimal Ni-B-Mo coating, with a uniform coating overall. The 3D image indicates small pits with a moderate depth, suggesting good bonding strength. Figure 17 shows the morphology of the No. 8 coating (Na_2_MoO_4_ = 5.6 g/L, NiCl_2_ = 25 g/L, t = 2 h) after heat treatment at 400 °C for 1 h, indicating that heat treatment may cause localized heating or phase transformation effects. The pits are more pronounced than in Figure 16, indicating increased delamination and reduced bonding strength. Figure 18 shows the morphology of the No. 12 coating (Na_2_MoO_4_ = 25.6 g/L, NiCl_2_ = 30 g/L, t = 4 h) under the same heat treatment conditions. The cracks were more pronounced, and even crack formation was observed, with severe delamination and the worst bonding strength. This suggests that higher concentrations of Na_2_MoO_4_ and NiCl_2_, as well as longer deposition times, may lead to the excessive internal growth of the coating, reducing its thermal stability. Overall, heat treatment at 400 °C for 1 h significantly impacted the stability of the coating. In the coatings, an excessive solution composition and prolonged deposition times could weaken the bonding strength. The optimal Ni-B-Mo coating on GCr15 steel (Na_2_MoO_4_ = 5.6 g/L, NiCl_2_ = 25 g/L, t = 2 h) maintained excellent bonding strength even after heat treatment.

#### 3.6.4. Microhardness Results and Analysis

**Figure 19:**
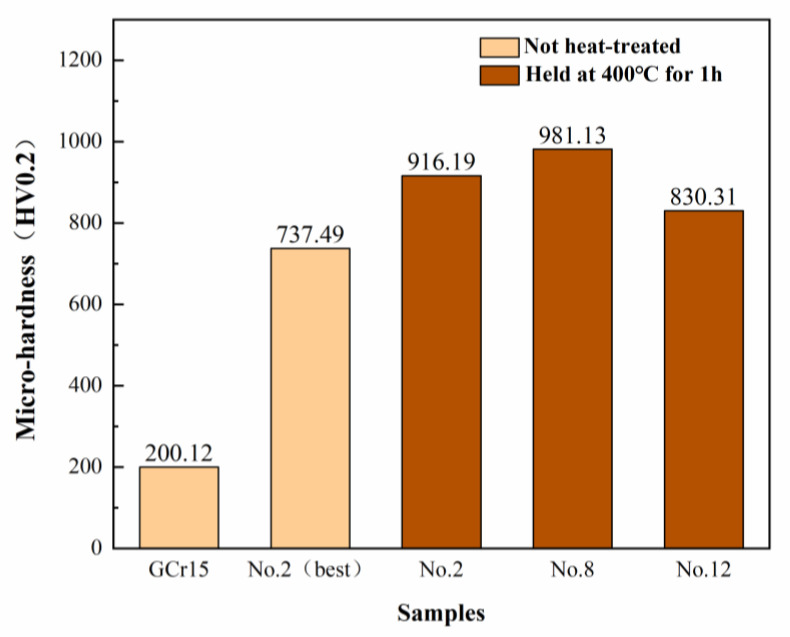
Microhardness of GCr15 substrate and Ni-B-Mo coating before and after heat treatment at 400 °C for 1 h.

Figure 19 shows the microhardness results for the GCr15 substrate and different Ni-B-Mo coatings before and after heat treatment at 400 °C for 1 h. The light-colored bars represent the hardness before heat treatment, while the dark brown bars represent the hardness after heat treatment at 400 °C for 1 h. According to the data presented in the figure, the hardness of the GCr15 substrate was the lowest at 200.12 HV_0_._2_, while the optimal Ni-B-Mo coating had a hardness of 737.49 HV0.2 before heat treatment. After heat treatment at 400 °C for 1 h with furnace cooling, the hardness significantly increased to 916.19 HV_0_._2_, indicating that heat treatment significantly enhanced the hardness of the Ni-B-Mo coating. The hardness of the No. 8 coating further increased to 981.13 HV0.2 after heat treatment, achieving the highest hardness among all the coatings. In contrast, the hardness of the No. 12 coating after heat treatment was 830.31 HV0.2, which was lower than that of the optimal Ni-B-Mo coating after heat treatment. This also confirms that excessively high concentrations of Na_2_MoO_4_ and NiCl_2_, as well as longer electroless plating times, still affect the effectiveness of hardness enhancement after heat treatment.

#### 3.6.5. Friction and Wear Performance Comparison and Analysis

**Figure 20:**
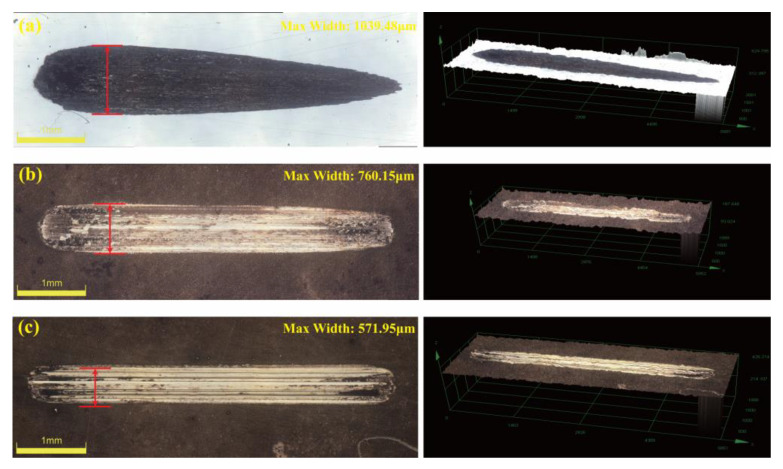
Friction and wear surface morphology after heat treatment: (**a**) GCr15 steel substrate; (**b**) optimal Ni-B-Mo coating; (**c**) optimal Ni-B-Mo coating heat-treated at 400 °C for 1 h.

Since the hardness of the coating after heat treatment was higher than that of the countermaterial GCr15 steel ball, Si3N4 ceramic balls were used as the countermaterial to further test the wear resistance of the coating. Figure 20 shows the 2D and 3D morphology of the friction and wear surfaces of GCr15 steel and the optimal Ni-B-Mo coating before and after heat treatment at 400 °C for 1 h. From the wear surface images, it can be observed that the maximum wear width of the GCr15 steel substrate was 1039.48 μm, with a rough surface and severe wear, indicating poor wear resistance. The wear width of the Ni-B-Mo coating was reduced to 760.15 μm, with a smoother surface, indicating an improvement in the wear resistance. After heat treatment at 400 °C for 1 h, the wear width of the coating further decreased to 571.95 μm. Three-dimensional morphology analysis further indicated that the wear of the GCr15 substrate was deeper, while the wear pits on the Ni-B-Mo coating were shallower, with the heat-treated coating surface being much smoother. It can be concluded that the wear resistance of the Ni-B-Mo coating can be further optimized after heat treatment at 400 °C for 1 h.

**Figure 21:**
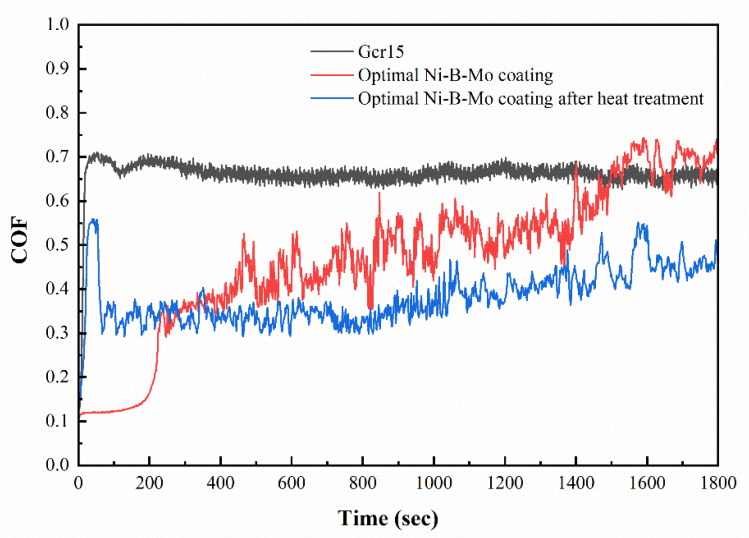
Comparison of friction coefficients of GCr15 steel substrate and optimal Ni-B-Mo coating before and after heat treatment.

Figure 21 shows the friction coefficient curves of GCr15 steel and the optimal Ni-B-Mo coating before and after heat treatment at 400 °C for 1 h. After a brief running-in period, the GCr15 steel reached a stable wear state, ultimately stabilizing at around 0.78. The optimal Ni-B-Mo coating showed a rapid decrease in the COF at around 200 s, stabilizing near 0.65. In contrast, the heat-treated optimal Ni-B-Mo coating reached a stable state more quickly, maintaining a lower COF value of approximately 0.35. Furthermore, after 200 s, the COF of the heat-treated Ni-B-Mo coating remained consistently lower than both that of the GCr15 steel and that of the non-heat-treated Ni-B-Mo coating.

**Figure 22:**
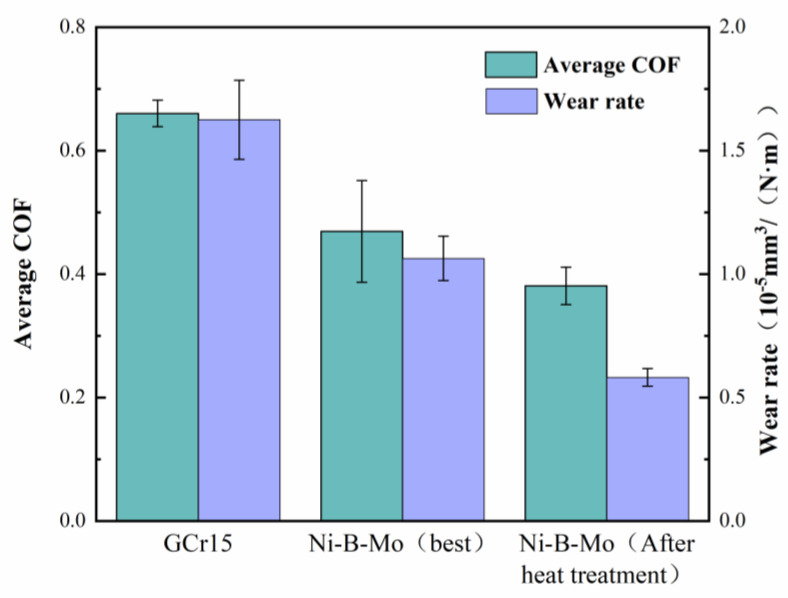
Average friction coefficient and wear rate of GCr15 steel substrate and optimal Ni-B-Mo coating before and after heat treatment.

Figure 22 shows the average friction coefficient and wear rate of the GCr15 steel substrate and the optimal Ni-B-Mo coating before and after heat treatment. The friction coefficients were 0.66018, 0.46918, and 0.381, respectively. The wear rates were 1.6252, 1.0632, and 0.5816 × 10^−5^ mm^3^/(N·m), respectively. The wear rate of the heat-treated optimal Ni-B-Mo coating was 64% and 45% lower than that of GCr15 steel and that of the non-heat-treated Ni-B-Mo coating, respectively. According to the Archard equation [34], the wear rate is inversely proportional to the hardness. After heat treatment, the Ni-B-Mo coating formed higher-hardness compounds on its surface, which reduced the surface wear during the friction process, resulting in the lowest wear rate of the heat-treated Ni-B-Mo coating. These results indicate that the optimized optimal Ni-B-Mo coating significantly improved the wear resistance of GCr15 steel, and the heat-treated coating further enhanced the wear resistance.

## 4. Conclusions

This study systematically investigated the effects of the Na_2_MoO_4_ and NiCl_2_ concentrations and deposition time on the properties of Ni-B-Mo electroless coatings on GCr15 steel. The main conclusions are summarized as follows:

(1) An appropriate concentration of Na_2_MoO_4_ (5.6 g/L) significantly promotes the co-deposition of Ni-B-Mo, improving both the deposition rate and coating performance. However, excessive Na_2_MoO_4_ (>15.6 g/L) decreases the deposition rate, induces embrittlement, and reduces the coating adhesion.
(2) A deposition time of 1–2 h maintains a relatively high deposition rate, while prolonged plating (>3 h) leads to bath depletion, a reduced deposition rate, and increased coating defects.
(3) With an increasing Na_2_MoO_4_ concentration, the coating surface morphology evolves from blackberry-like and fish egg-like structures to cauliflower-like and broccoli-like structures.
(4) An NiCl_2_ concentration in the range of 20–30 g/L has a relatively minor effect on the deposition rate and coating properties, primarily acting to stabilize the Ni^2+^ supply and improve the coating compactness.
(5) Based on orthogonal experimental analysis, the optimal plating parameters were determined as follows: Na_2_MoO_4_ = 5.6 g/L, NiCl_2_ = 25 g/L, deposition time = 2 h, pH > 13, temperature = 90 °C, and stirring speed = 100 rpm. Under these conditions, the coating exhibited high hardness, excellent adhesion, and superior wear resistance.
(6) Post-deposition annealing at 400 °C for 1 h transformed the coating structure from amorphous to nanocrystalline (Ni_2_B, Ni_3_B, and Mo_2_C phases), increasing the hardness from 737.49 HV to 916.19 HV. The friction coefficient decreased from 0.66 to 0.38, and the wear rate was reduced by 64% compared to that of the GCr15 steel substrate.

In conclusion, the optimized Ni-B-Mo coating for GCr15 steel demonstrates outstanding mechanical and wear-resistant properties, providing technical support for the application of high-performance wear-resistant coatings.

## Figures and Tables

**Table 1:** GCr15 steel’s chemical composition (wt%).

C	Cr	Mn	Si	Ni	Cu	P	S	O
0.95	1.44	0.36	0.27	0.06	0.07	0.13	0.004	0.0004

**Table 2:** Composition and process parameters of the electroless Ni-B-Mo plating solution.

Chemical Composition and Process Conditions	Concentration (g/L) and Parameters
NiCl_2_·6H_2_O (nickel chloride hexahydrate)	20~30
NaBH_4_ (sodium borohydride)	1
C_2_H_8_N_2_ (ethylenediamine)	60
Na_2_MoO_4_ (sodium molybdate)	0.6~25.6
Pb(NO_3_)_2_ (lead nitrate)	0.03
NaOH (sodium hydroxide)	40
PH	>13
Deposition time (h)	1~4
Stirring speed (rpm)	100
Temperature (°C)	90

**Table 3:** Orthogonal experiment.

Experimental Group	Na_2_MoO_4_ (g/L)	t (h)	NiCl_2_ (g/L)
1	0.6	1	20
2	5.6	2	25
3	10.6	3	30
4	15.6	4	20
5	20.6	1	25
6	25.6	2	30
7	0.6	3	20
8	5.6	4	25
9	10.6	1	30
10	15.6	2	20
11	20.6	3	25
12	25.6	4	30

**Table 4:** Deposition rate (μm/h) of each group in the Ni-B-Mo chemical plating orthogonal experiment.

Serial No.	1	2	3	4	5	6
Deposition Rate (μm/h)	16.75	15.21	13.49	110.36	12.28	10.59
Serial No.	7	8	9	10	11	12
Deposition Rate (μm/h)	11.26	9.35	16.75	8.24	9.49	6.91

**Table 5:** Range analysis results for the deposition rate in the Ni-B-Mo chemical plating orthogonal experiment.

	Factor		Sodium Molybdate (g/L)	Time (h)	Nickel Chloride (g/L)
Evaluation Index					
Deposition Rate (μm/h)		K1	28.01	45.78	48.95
		K2	24.56	34.03	46.33
		K3	30.24	34.24	45.38
		K4	20.94	26.62	
		K5	21.77		
		K6	15.14		
		$\bar{K1}$	14.00	15.26	12.24
		$\bar{K2}$	12.28	11.34	11.58
		$\bar{K3}$	15.12	11.41	11.35
		$\bar{K4}$	10.47	8.87	
		$\bar{K5}$	10.89		
		$\bar{K6}$	7.57		
		R (Range)	7.55	6.39	0.89
Primary and Secondary Factors			ABC		
Optimal Combination			A_3_B_1_C_1_		

**Table 6:** Microhardness (HV_0_._2_) in Ni-B-Mo electroless plating orthogonal experiment.

Serial No.	1	2	3	4	5	6
Hardness	881.00	737.49	727.76	814.08	582.30	745.51
Serial No.	7	8	9	10	11	12
Hardness	783.91	771.94	699.61	755.57	792.67	730.34

**Table 7:** Range analysis of microhardness in Ni-B-Mo electroless plating orthogonal experiment.

	Factor		Sodium Molybdate (g/L)	Time (h)	Nickel Chloride (g/L)
Evaluation Index					
Hardness (HV0.2)		K1	1664.90	2162.91	3234.55
		K2	1509.43	2238.57	2884.40
		K3	1427.37	2304.34	2903.22
		K4	1569.65	2316.36	
		K5	1374.97		
		K6	1475.85		
		$\bar{K1}$	832.45	720.97	808.64
		$\bar{K2}$	754.71	746.19	721.10
		$\bar{K3}$	713.69	768.11	725.81
		$\bar{K4}$	784.83	772.12	
		$\bar{K5}$	687.48		
		$\bar{K6}$	737.93		
		R (Range)	144.97	51.15	87.54
Primary and Secondary Factors			ACB		
Optimal Combination			A_1_B_4_C_1_		

**Table 8:** Optimal electroless Ni-B-Mo plating parameters for GCr15 steel.

Chemical Composition and Process Conditions	Concentration (g/L) and Parameters
NiCl_2_·6H_2_O (nickel chloride hexahydrate)	25
NaBH_4_ (sodium borohydride)	1
C_2_H_8_N_2_ (ethylenediamine)	60
Na_2_MoO_4_ (sodium molybdate)	5.6
Pb(NO_3_)_2_ (lead nitrate)	0.03
NaOH (sodium hydroxide)	40
PH	>13
Deposition time (h)	2
Stirring speed (rpm)	100
Temperature (°C)	90

## Data Availability

The original contributions presented in the study are included in the article, further inquiries can be directed to the corresponding author.
